# Central Nervous System Vasculitis for Cryptococcosis in an Immunocompetent Patient

**DOI:** 10.3390/diseases6030075

**Published:** 2018-08-31

**Authors:** Dan Zimelewicz Oberman, Liliana Patrucco, Carolina Cuello Oderiz

**Affiliations:** 1 University Institute, Hospital Italiano de Buenos Aires, Potosí 1199, Argentina; 2 Department of Neurology, Hospital Italiano de Buenos Aires, Potosí 1199, Argentina; liliana.patrucco@hospitalitaliano.org.ar (L.P.); carolinacuellooderiz@gmail.com (C.C.O.)

**Keywords:** angiography, cryptococcosis, immunocompetent, meningoencephalitis, vasculitis

## Abstract

Cryptococcal meningitis is a life-threatening condition caused by a fungal pathogen, *Cryptococcus neoformans*, that can infect both immunosuppressed and immunocompetent hosts. It is an important cause of morbidity and mortality in severely immunodeficient patients. However, in an immunocompetent patient it represents a diagnostic challenge, mainly because it is extremely rare, but also because of its nonspecific clinical manifestation. Neurovascular involvement in cryptococcal meningitis is rare and not well known and only few reports have described this association. We describe a cryptococcal meningitis in an immunocompetent patient associated with central nervous system vasculitis.

## 1. Introduction

Cryptococcal meningitis (CM) is a life-threatening condition caused by a fungal pathogen, *Cryptococcus neoformans*, that can infect both immunosuppressed and immunocompetent hosts. The infection of the central nervous system (CNS) is the most frequent form presented of extrapulmonary disease, which can result in high morbidity and mortality [1,2].

Some reports revealed that 10–40% of Human immunodeficiency virus (HIV)-negative patients with CM have no apparent immune deficiency, such as diabetes, cancer, chemotherapy and autoimmune diseases (mainly systemic lupus erythematosus and sarcoidosis) [3].

Cryptococcosis is an opportunistic fungal infection that affects the CNS, mostly in immunosuppression patients [4]. It rarely affects immunocompetent patients, but when it does, the most clinical presentation is localized disease [5,6]. 

Individuals with CM can occasionally present small vessel vasculitis causing infarcts primarily in the basal ganglia, internal capsule and thalamus [7]. However, only few reports have described neurovascular involvement in CM, especially in immunocompetent patients [8,9]. It is therefore not well known the how often neurovascular involvement is present.

CM seldom occurs in those without compromised immune systems and vasculitis is a rare clinical manifestation. Here we report a case of CNS cryptococcosis in an immunocompetent patient with vasculitis.

## 2. Case Report

A 35-year-old woman with a history of having lived near a pigeon farm, with erosive esophagitis, anorexia nervosa, malnutrition (IMC = 17.08 Kg/m^2^) (reference IMC = 18.5–24.99 Kg/m^2^) and a family history of rheumatoid arthritis, was admitted to our hospital complaining of one month of progressive neurological symptoms: holocranial headache, vomiting, blurred vision, bradypsychia, vertigo, aphasia, gait instability and right-sided paresthesia and weakness. During the admission, the patient was somnolent, febrile and presented a generalized tonic-clonic seizure. Physical examination showed deep tendon reflex preserved, isoreactive pupils, bilateral horizontal nystagmus, severe right-sided paresis, bilateral ataxia with right-sided predominance and meningeal stiffness. 

A lumbar puncture (LP) showed an elevated open pressure at 35 cm H_2_O (reference range, <20 cm). Cerebrospinal fluid (CSF) analysis revealed 271/mm^3^ cells (reference range, 0 to 10/mm^3^), with 95% mononuclear cell, which were predominantly lymphocytes. The glucose level was 7 mg/dL (reference range, 40 to 70 mg/dL) and protein level was 168 mg/dL (reference range, 15 to 45 mg/dL). Polymerase chain reaction (PCR) for Herpes simplex, Epstein barr, Varicella, Enterovirus, Tuberculosis and atypical Mycobacteria were negative. India ink preparation was positive and cryptococcal antigen showed positive results at 1/100. In order to rule out a CNS lymphoma as a cause of predominant lymphocytes seen in the CSF, we performed flow cytometry which did not show clonality. Treatment was initiated with liposomal amphotericin B 0.63 mg/kg/day, flucytosine 100 mg/kg levetiracetam 500 mg bid and medprednisone 1 mg/kg/day. Five LPs evacuations were made due to neurological deterioration. Mycological culture was positive for *Cryptococcus neoformans* variant. 

An extensive analysis to rule out immunocompromised status was performed. We aimed to discard hepatic insufficiency, chronic renal disease, immunodeficiency associated with pregnancy, IgA deficiency, HIV, idiopathic deficiency of CD4, autoimmune rheumatic diseases, lymphoma and other neoplasias. All the results were normal. Cancer screening was done with a pan-tomographic exam (Hospital Italiano, Buenos Aires, Argentina) and serologic exams (Hospital Italiano, Buenos Aires, Argentina): Alpha-fetoprotein (AFP), carcinoembryonic antigen (CEA) and CA-125, 19.9, 15.3 and 6.2, which also were negative.

**Figure 1:**
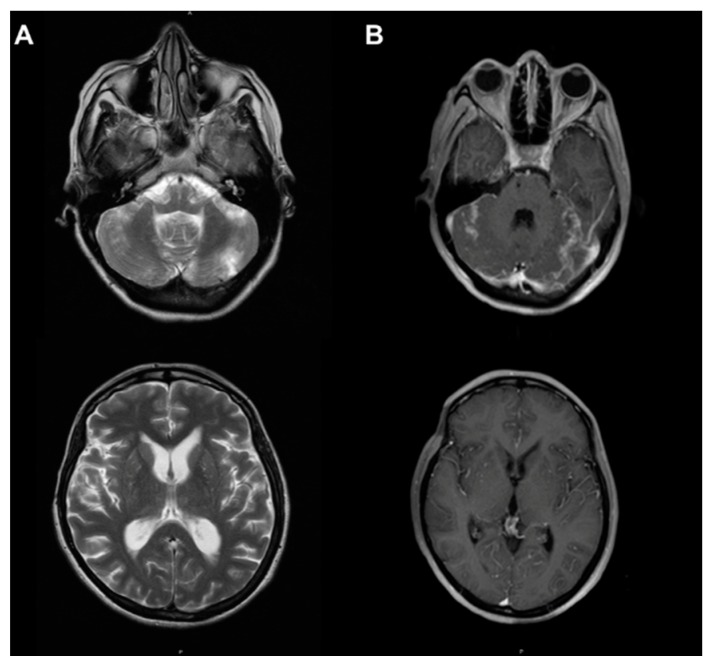
Axial magnetic resonance with contrast. (**A**) T2 sequence: hyperintense bilateral cerebellar lesions and in the right cingulate gyrus. (**B**) T1 with gadolinium: demonstrating gadolinium uptake in the posterior cranial fossa.

Magnetic resonance imaging (MRI, Hospital Italiano, Buenos Aires, Argentina) showed hydrocephalus, bilateral cerebellar lesions with increased signal in T2-weighted and fluid attenuation inversion recovery (FLAIR) sequences. Also, we observed a focal lesion on the right-side gyrus of the cingulum in relation to the peak of the corpus callosum (Figure 1). Cerebellar lesions showed an evolution in time of ischemic vascular type, which could correspond to vasculitis.

**Figure 2:**
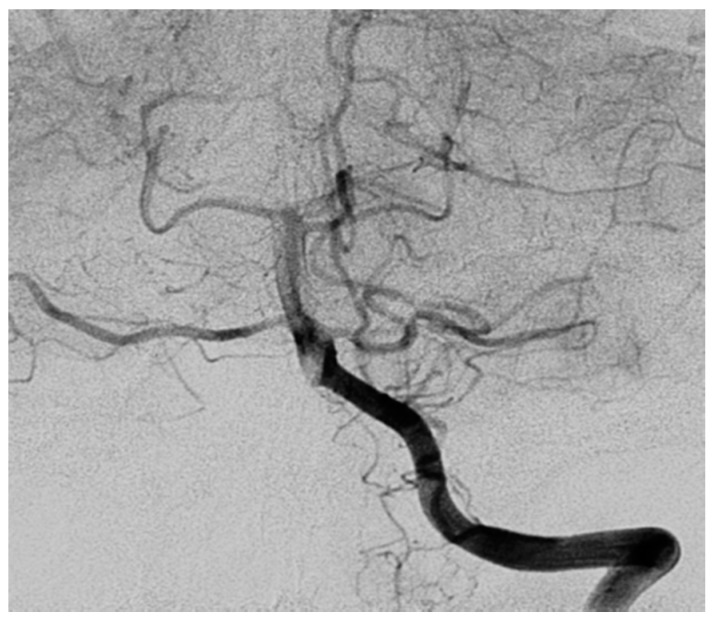
Digital subtraction angiography demonstrating right vertebral artery: changes in the caliber of the vessels of the right antero-inferior cerebellar artery as well as the tonsillar branch of the right postero-inferior cerebellar artery.

Digital subtraction angiography (Hospital Italiano, Buenos Aires, Argentina) was performed and showed changes in the caliber of the vessels of the right antero-inferior cerebellar artery as well as the tonsillar branch of the right postero-inferior cerebellar artery (Figure 2). Similar findings were seen in the right parietal region in cortical branches. This procedure confirmed CNS vasculitis.

The patient started with diplopia and important visual deficit. Funduscopic exam revealed both pink papillae with net borders. Visual evoked potential showed prolonged latency of the p100 wave. It was interpreted as bilateral optic neuropathy and intracranial hypertension secondary to cryptococcal infection. After 30 days of treatment with amphotericin B and flucytosine, it was changed for fluconazole 400 mg for 10 weeks more, with good clinical response.

After a one-year follow-up, the patient presented an expressive improvement of laboratory and the physical examination showed mild ataxia, tinnitus and mild right hypoacusia. However, the patient presented diminished bilateral visual acuity and hydrocephalus. Vestibular myogenic evoked potentials showed involvement of the vestibular-spinal pathway with right-sided predominance. The patient is now on vestibular rehabilitation and is being evaluated for a cochlear implant.

## 3. Discussion

Cryptococcosis affecting immunocompetent patients represents a diagnostic challenge, mainly because it is extremely rare, but also because of it nonspecific clinical manifestations. Consequently, delay in the diagnosis and in the onset of specific treatment is frequently observed [10,11].

Headache is the most common symptom (85%) and can be preceded by other manifestations for days to weeks [12]. Lumbar puncture should be immediately performed in cases of suspicion, with measurement of intracranial pressure. 

In the present case, MRI showed an uptake in posterior cranial fossa in T1 and bilateral hyperintense lesions in the cerebellar hemisphere and in the right cingulate lobe in T2. Digital angiography revealed bilateral atypical vasculitis in the posterior cranial fossa.

Although *Cryptococcus neoformans* infection has high affinity for the CNS, it rarely results in cerebral infarction, when compared with other infections [13]. Previously studies reported some infections produce vasculitis of the CNS, such as Cytomegalovirus, Herpes zoster and Tuberculosis [14]. These infections were discharged through LP and PCR analyses. Several mechanisms are involved in cerebral vasculitis which can produce inflammation, vasospasm, constriction, eventually thrombosis and occlusion, being a different diagnosis to stroke mimic [15]. Some reports revealed CM associated to vasculitis as having a poor prognosis when compared to patients without cerebral infarcts [16]. This suggests that vascular injury plays a role in the outcome of patients with cryptococcal meningitis. Nevertheless, the role of CM in vasculopathy is uncertain [6].

The patient presented with important visual deficit and intracranial hypertension. Adjuvant treatment with steroids and regular lumbar drainages was initiated. Antifungal therapy coupled with earlier and aggressive treatment of intracranial hypertension remain the mainstay of treatment of visual loss in patients with CM [17]. However, the above findings are not seen in most cases of cryptococcosis in immunocompetent hosts [18].

CNS cryptococcosis rarely occurs in those without compromised immune systems. However, the patient was anorexic with reduced IMC, which could affect her immunity system. There is evidence supporting that patients with malnutrition could have their immunity influenced and it is considered one of the most relevant risk factors for illness. Opportunistic infections in patients with poor nutrition and IMC alteration should be considered for investigation [19].

In regard to pharmacological, the mainstays of treatment of CM include standard antifungal therapy, managing of life-threatening conditions and treatment of underlying conditions. As to antifungal therapy amphotericin B (0.7 mg/kg/day) and flucytosine, followed by consolidation and maintenance of fluconazole is the mainstays treatment of CM [20,21]. The patient was treated with liposomal amphotericin B and flucytosine followed by fluconazole for 10 weeks. Serial LPs were required for symptomatic relief of intracranial hypertension. Observation during the inpatient stay showed low potassium levels, as a consequence of amphotericin B administration. 

## 4. Conclusions

Cryptococcal meningitis is a rare opportunistic disease, especially associated with central nervous system vasculitis in immunocompetent patients. The nonspecific manifestations of this condition and its subacute or chronic onset usually leads to delayed identification and treatment. The diagnosis is very challenging and usually relies on lumbar puncture and cultures.

Patients with malnutrition should be investigated for atypical infection, such as cryptococcal meningitis. CNS vasculitis associated with cryptococcal infection is rare and not well described. This infection should be investigated and treated correctly to avoid neurological complications.
